# Safety and Pharmacokinetic Profiles of Long-Acting Injectable Antiretroviral Drugs for HIV-1 Pre-Exposure Prophylaxis: A Systematic Review and Meta-analysis of Randomized Trials

**DOI:** 10.3389/fphar.2021.664875

**Published:** 2021-07-07

**Authors:** Gilbert Lazarus, Vincent Kharisma Wangsaputra, Christianto, Melva Louisa, Vivian Soetikno, Raph L. Hamers

**Affiliations:** ^1^ Faculty of Medicine, Universitas Indonesia, Jakarta, Indonesia; ^2^ Department of Pharmacology and Therapeutics, Faculty of Medicine, Universitas Indonesia, Jakarta, Indonesia; ^3^ Eijkman-Oxford Clinical Research Unit, Jakarta, Indonesia; ^4^ Centre for Tropical Medicine and Global Health, Nuffield Department of Medicine, University of Oxford, Oxford, United Kingdom

**Keywords:** cabotegravir, long acting injectable (LAI), HIV-human immunodeficiency virus, pre-exposure (PrEP) prophylaxis, rilpivirine

## Abstract

**Objectives:** To investigate the safety and pharmacokinetic profiles of long-acting injectable pre-exposure prophylaxis (LAI PrEP), notably cabotegravir (CAB-LA) and rilpivirine (RPV-LA), for the prevention of human immunodeficiency virus-1 (HIV-1) infection.

**Methods:** Eligible randomized trials of LAI PrEP in HIV-uninfected and/or healthy patients were included and assessed with the Revised Cochrane risk-of-bias tool for randomized trials. Where feasible, a meta-analysis was performed for safety outcomes by using a random-effects model with risk ratios and their 95% confidence intervals as the common effect measure. The protocol was registered with PROSPERO CRD42020154772.

**Results:** Eight studies cumulating a total of 666 participants were included in this systematic review, including five (362 intervention-arm volunteers) and four trials (194 intervention-arm volunteers) that investigated CAB-LA and RPV-LA, respectively. We found that both CAB-LA and RPV-LA were generally well-tolerated as their safety profiles were similar to placebo in terms of any adverse event (AE), serious AE, and AE-related withdrawals. Furthermore, pharmacokinetic analyses revealed favorable prospects in viral inhibitory activity of CAB-LA and RPV-LA. Intramuscular (IM) injection of CAB-LA 600 mg Q8W was superior to CAB-LA 800 mg Q12W in male participants, while the same was true for RPV-LA 1200 mg IM Q8W over other dosing regimens. Although these results are promising, further research is required to confirm the findings on RPV-LA as current evidence is limited.

**Conclusion:** CAB-LA and RPV-LA have promising safety and pharmacokinetic profiles. The preventive efficacy of these agents is being evaluated in Phase 3 trials.

## Introduction

Human immunodeficiency virus-1 (HIV-1) infection remains a major global health issue with over 39 million deaths to date and more than 36 million people currently living with HIV-1 (Pandey and Galvani, 2019). Continuous transmission occurs through sexual intercourse and parenteral exposure (Patel et al., 2014). Several breakthroughs to alleviate these burdens have been made during the past few years. Antiretroviral (ARV) drugs have emerged as a potential tool for preventing HIV-1 transmission when used by individuals at risk for HIV infection as an oral pre-exposure prophylaxis (PrEP) (Lundgren and Phillips, 2018). However, because of concerns related to the requirement for high levels of patients’ adherence to these daily consumed agents (Sidebottom et al., 2018), further innovations are warranted to minimize the risk of non-adherence and maximize the potential of PrEP.

Recently, long-acting injectable pre-exposure prophylaxis (LAI PrEP) has emerged as a potential solution. These agents may provide long-term protection to HIV-susceptible populations through multi-monthly injections, thus reducing the risk of non-adherence by establishing a slow-release drug depot to prevent HIV-1 infection (Kerrigan et al., 2018; Clement et al., 2020). Recent studies have focused on establishing the optimal dosing strategies to maximize the effectiveness of LAI PrEP, notably cabotegravir (CAB-LA) and rilpivirine (RPV-LA), which are the leading candidates of long-acting HIV PrEP (Markowitz et al., 2017; Nyaku et al., 2017). Therefore, this systematic review aims to summarize the available evidence on safety and pharmacokinetic profiles of antiretroviral drugs investigated as LAI PrEP, notably CAB-LA and RPV-LA, in order to find the optimal dosing strategies.

## Materials and Methods

This systematic review was conducted in accordance with the Cochrane Handbook for Systematic Reviews of Intervention version 6 (Higgins et al., 2019) and reported in accordance with the Preferred Reporting Items for Systematic Reviews and Meta-Analyses (PRISMA) statement (Moher et al., 2009). A detailed protocol has been prospectively registered in PROSPERO [CRD42020154772 (Lazarus and Christianto, 2020)]. The deviations from the protocol are summarized on Supplementary Table S1.

### Search Strategy

We searched the literature for eligible studies published from inception up to November 2020 through PubMed, Scopus, Cumulative Index to Nursing and Allied Health Literature (CINAHL), EBSCO MEDLINE, and Cochrane Controlled Register of Trials (CENTRAL) databases. Additionally, ProQuest and Google Scholar databases were screened for gray literature and manual searches were performed by hand-searching reference lists of included studies and previous reviews. Searches were conducted by two independent investigators (GL and C) using keywords listed on Supplementary Table S2, and any discrepancies were resolved by a third investigator (RLH)–also in blinded fashion. Any studies judged potentially eligible from title and abstract screening by either reviewer were retrieved for full-text assessments. No language restrictions were applied upon title and abstract screening.

### Study Eligibility Criteria

Inclusion criteria were set as the following: 1) study design, randomized trials; 2) study population, HIV-uninfected and/or healthy patients; 3) intervention, LAI PrEP (e.g., CAB-LA or RPV-LA), and 4) outcomes, including outcomes of efficacy, safety, tolerability, and pharmacokinetic profiles of LAI PrEP. Conversely, exclusion criteria were set to filter out irretrievable full-text articles or studies not in English.

Since the full-text articles of studies evaluating the efficacy of LAI PrEP (i.e., HPTN 083 (HIV Prevention Trials Network, 2020a) and HPTN 084 (HIV Prevention Trials Network, 2020b) have yet to be published, this review primarily investigated the safety and pharmacokinetics profiles of LAI PrEP. The primary safety outcome of this review was the frequency of patients experiencing adverse events (AE) grade ≥≥2–as defined by the corrected version 2.1 of Division of AIDS (DAIDS) Table for Grading the Severity of Adult and Pediatric Adverse Events (National Institute of Allergy and Infectious Diseases, 2017a), while the secondary safety outcomes include frequency of patients: 1) experiencing any AE, 2) experiencing serious AE, and 3) withdrawing treatment due to AE. Pharmacokinetic parameters investigated in this study included C_max_, T_max_, Cτ, AUC_0-_τ, AUC_0–∞_, t_1/2,_ CL/F, and proportion of patients with plasma drug concentration of >4 × 90% protein-adjusted inhibitory concentration (PA-IC_90;_ see Definitions in Supplementary Material) –which was the concentration deemed satisfactory to provide adequate protection. The 4x PA-IC_90_ value of CAB-LA was set at 0.664 μg/ml (Markowitz et al., 2017; Landovitz et al., 2018b), while that of RPV-LA was set at 50 ng/ml (Bekker et al., 2020).

### Data Extraction and Risk of Bias Assessment

The following relevant data from included studies were extracted: 1) first author’s or trial’s names and trial identifiers; 2) study characteristics, including recruitment period, study design, interventions and comparators, and follow-up period; 3) subject characteristics, i.e., sample size, mean age, frequency and proportion of female population; and 4) outcomes.

Risk of bias assessment of included trials were performed by using the Revised Cochrane risk-of-bias tool for randomized trials ver. 2.0. (RoB2)–consisting of five bias domains: randomization, assignment and adhering to intervention, missing outcome data, measurement of the outcome, and selection of reported results. Risk of bias assessment results were judged to be low, unclear/some concerns, or high (Sterne et al., 2019). Data of included trials were extracted by using a pre-specified form and managed by using the MS Excel^®^ for Office 365 MSO ver. 2002 (Microsoft Corporation, Redmond, WA, United States, 2018). Data extraction and risk of bias assessment were conducted by two independent reviewers (GL and VK, GL, and Christianto), and any discrepancies were resolved by consensus with a third reviewer (ML or RLH). Details on risk of bias assessments are shown on Supplementary Figure S1.

### Statistical Analysis

All statistical analyses were performed using R ver. 4.0.0 (R Foundation for Statistical Computing, Vienna, Austria) (R Core Team, 2020) with the additional *meta* (ver. 4.9–6) (Balduzzi et al., 2019), *ggplot2* (ver. 3.3.2) (Wickham, 2016), and *robvis* (ver. 0.3.0) (McGuinness and Higgins, 2020) packages. Dichotomous outcomes were presented as frequency and proportion, while continuous data were presented as geometric mean and 95% confidence interval (CI) or between-person coefficient of variability (%CV).

Due to differences in dosing strategy and follow-up period, meta-analysis was conducted only for safety outcomes using random-effects model with risk ratios (RRs) as the common effect measure. Furthermore, meta-analysis was conducted only when heterogeneity do not pose imminent threats to outcome validity (*I* ^2^ < 75% or *p* > 0.01). Heterogeneity between studies was investigated using the Cochran Q test and I^2^ statistics, where heterogeneity was classified as negligible (0–25%), low (25–50%), moderate (50–75%), and high (>75%) to *I* ^2^ values of 0–25, 25–50, 50–75, and >75%, respectively. The significance level for Cochran Q test was set at 10%. If feasible, subgroup analysis (if *n* ≥ 2 in both subgroups) was conducted to identify potential sources of heterogeneity based on risk of bias and masking status, while sensitivity analysis was performed through leave-one-out analysis and simultaneous exclusion of studies with high risk-of-bias. Publication bias was assessed only when sufficient number of studies were present (*n* ≥ 10) (Higgins and Green, 2011) using funnel plot and Egger’s (Egger et al., 1997) and Begg’s (Begg and Mazumdar, 1994) tests.

## Results

### Study Selection and Characteristics

**FIGURE 1:**
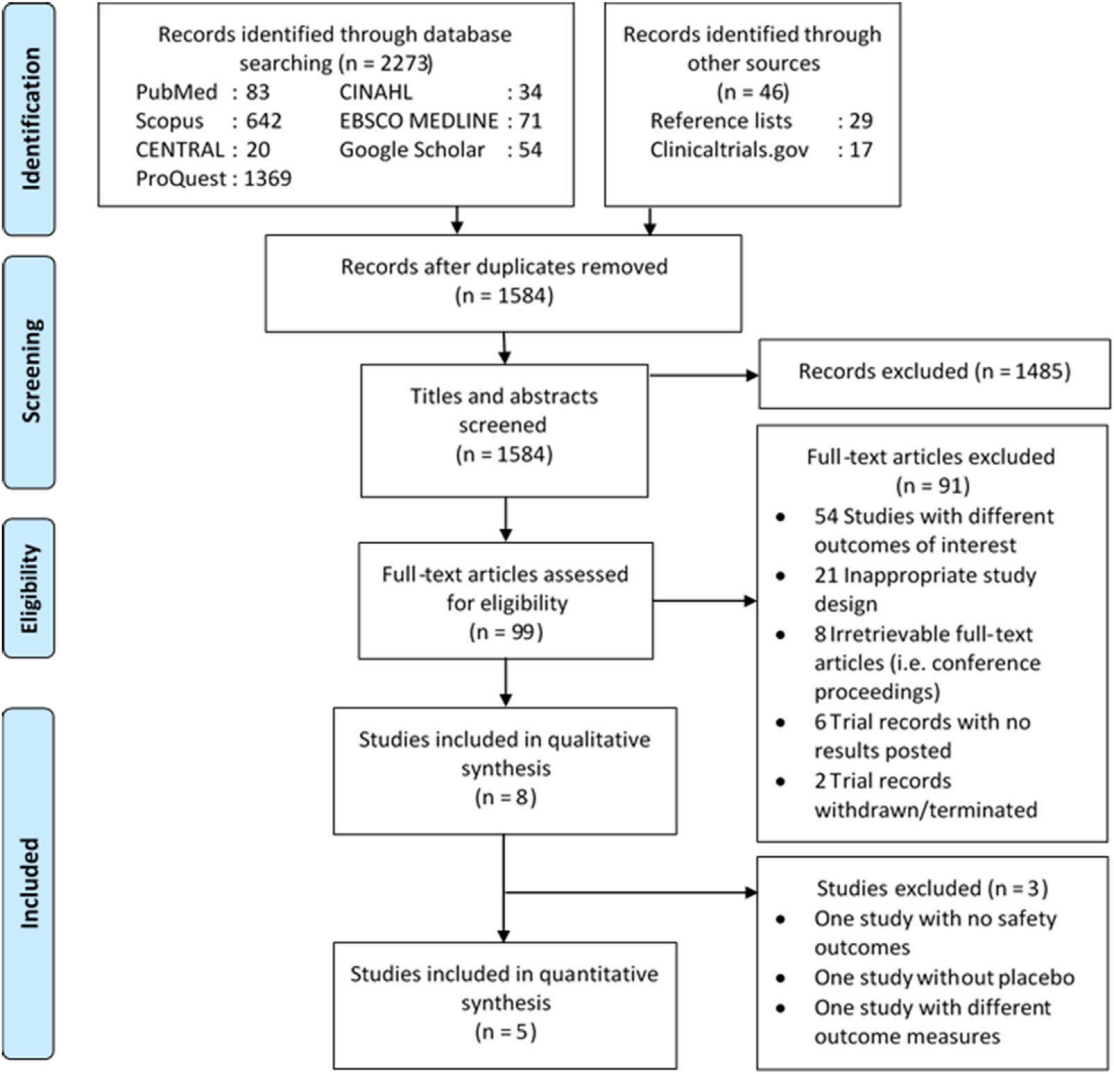
Diagram flow illustrating literature search process. CENTRAL, Cochrane Central Register of Controlled Trials; CINAHL, Cumulative Index to Nursing and Allied Health Literature.

Details on the literature search process are shown in Figure 1. The initial search yielded 2,319 records, of which 735 were deduplicated and 1,485 were excluded following title and abstract screening. The remaining 91 records were excluded due to different outcomes of interest (54 records), inappropriate design (21 records), irretrievable full-text articles (eight records), and ineligible trial records (eight records), resulting in the inclusion of eight studies (seven unique randomized trials) (Jackson et al., 2014; Spreen et al., 2014a; Spreen et al., 2014b; Verloes et al., 2015; Markowitz et al., 2017; Landovitz et al., 2018b, Landovitz R. J. et al., 2020; Bekker et al., 2020). All but one irretrievable record (Landovitz, 2020) were conference proceedings, for which full-text articles have been included in this review. Lastly, five studies qualified for quantitative analysis as three studies were excluded due to different outcome measures (i.e., incidence per 100 person-years) (Landovitz R. J. et al., 2020), no placebo (Spreen et al., 2014b), and no safety outcomes (Jackson et al., 2014).

Among the included trials, four were phase 1 (Jackson et al., 2014; Spreen et al., 2014a; Spreen et al., 2014b; Verloes et al., 2015) and three were phase 2 studies (Markowitz et al., 2017; Landovitz et al., 2018b; Bekker et al., 2020; Landovitz R. J. et al., 2020). Four were multi-centered (Spreen et al., 2014b; Markowitz et al., 2017; Landovitz et al., 2018b; Bekker et al., 2020; Landovitz R. J. et al., 2020; ) and three were single-centered trials (Jackson et al., 2014; Spreen et al., 2014a; Verloes et al., 2015). Risk of bias assessment resulted in low risk for five studies (Jackson et al., 2014; Verloes et al., 2015; Markowitz et al., 2017; Landovitz et al., 2018b; Bekker et al., 2020), moderate risk for one study (Spreen et al., 2014b), and high risk for two studies (Spreen et al., 2014a; Landovitz R. J. et al., 2020) (Supplementary Figure S1). The tail-phase of HPTN 077 trial (Landovitz R. J. et al., 2020) was judged to have high risk of bias due to prevalent loss to follow-up, whereas the other two studies (Spreen et al., 2014a; Spreen et al., 2014b) had moderate-to-high risk of bias due to unclear assignment and adherence to interventions (Supplementary Table S3). In addition, the study by Spreen et al. (2014a) had an unclear risk of bias on the outcome ascertainment domain, thus rendering the study high risk of bias (Supplementary Figure S2).

A total of 666 participants (384 female participants [57.7%]) were included in the meta-analysis, of which 362 received CAB-LA and 194 received RPV-LA (Table 1). Two cohorts (20 participants) enrolled in the study by Spreen et al. received concomitant CAB-LA and RPV-LA (Spreen et al., 2014b). CAB-LA was administered in two routes (i.e., intramuscular [IM] and subcutaneous [SC]), with doses ranging from 100 to 800 mg and intervals ranging from 4 to 12 weeks. RPV-LA was only administered intramuscularly with doses ranging from 300 to 1,200 mg and intervals ranging from 4 to 8 weeks.

**TABLE 1:** Characteristics of included studies and participants.

Author/Trial name (NCT ID); year	Recruitment period	Study characteristics				Subject characteristics			Follow-up period (weeks)	RoB2 score^b^
		Phase	Masking	Location	Treatment	Sample size	Age (years)^a^	Male; *n* (%)		
Cabotegravir LA										
HPTN 077 (tail phase; NCT02178800) Landovitz et al. (2018a); 2020	Feb 9, 2015–May 27, 2016	2a	Double-blind	Multicenter	CAB-LA IM 600 mg Q8W vs. placebo	177 (134 I, 43 C)	31 (24–39)	117 (66.1)	52–76	**+**
HPTN 077 (NCT02178800) (Landovitz et al. (2018b); 2018					CAB-LA IM 800 mg Q12W vs. placebo	199 (151 I, 48 C)	31 (24–39)	132 (66.3)	52–76	−
ECLAIR (NCT02076178) Markowitz et al. (2017); 2017	Mar 27, 2014–Feb 23, 2016	2a	Double-blind	Multicenter	CAB-LA IM 800 mg Q12W + placebo	127 (106 I, 21 C)	31 (range: 20–61)	0 (0.0)	81	−
Spreen et al. (NCT01756131) Spreen et al. (2014a); 2014	NR	1	Open label	Single center	CAB-LA IM 100, 200, 200 × 2, 400, 400 × 2 mg single-dose vs. placebo	72 (58 I, 14 C)	35.1 ± 10.4	33 (45.8)	12–52	+
					CAB-LA SC 100, 200, 400 mg single-dose vs. placebo					
Spreen et al. (NCT01593046) Spreen et al. (2014b); 2014	May 31, 2012–Dec 19, 2013	1	Open label	Multicenter	CAB-LA IM 800/SC 200 × 3 mg Q4W	47	39.5 ± 13.9	17 (36.2)	52	?
					CAB-LA IM 800/IM 200 × 3 Q4W + RPV-LA IM 1200/900 mg					
					CAB-LA IM 800/IM 400 × 3 Q4W + RPV-LA IM 1200/900 mg					
					CAB-LA IM 800 mg Q12W					
Rilpivirine LA										
HPTN 076 (NCT02165202) Bekker et al. (2020); 2020	Apr 13, 2015–Feb 27, 2017	2	Double-blind	Multicenter	RPV-LA IM 1200 mg Q8W	136 (91 I, 45 C)	31 (25–38)	136 (100)	76	−
Verloes et al. (NCT01031589) Verloes et al. (2015); 2015	Jan 21, 2010–Jul 19, 2011	1	Open label	Single center	RPV-LA IM 300, 600 mg single-dose	11	47 (range: 31–58)	6 (31.6)	12–24	−
			Double-blind		RPV-LA IM 1200/600/600 mg Q4W vs. placebo	8 (6 I, 2 C)				
Jackson et al. (NCT01275443) Jackson et al. (2014); 2014	Jan 27, 2011–Aug 3, 2012	1	Open label	Single center	RPV-LA IM 300, 600, 1,200 mg	66	35.1 ± 9.2	60 (90.9)	12	−

a Unless specified, age is presented in mean ± standard deviation (SD) or median (interquartile range).

b Assessed using the Revised Cochrane risk-of-bias tool for randomized trials ver. 2.0 (RoB 2) (Sterne et al., 2019); −, low risk; ?, some concerns; **+**, high risk. C, control group; CAB, cabotegravir; I, intervention group; IM, intramuscular; LA, long acting; NCT ID, Clinicaltrials.gov identifier; RoB2, Revised Cochrane risk-of-bias tool for randomized trials; RPV, rilpivirine; SC, subcutaneous; Q4W, every 4 weeks; Q8W, every 8 weeks; Q12W, every 12 weeks.

### Outcomes

#### Safety

**FIGURE 2:**
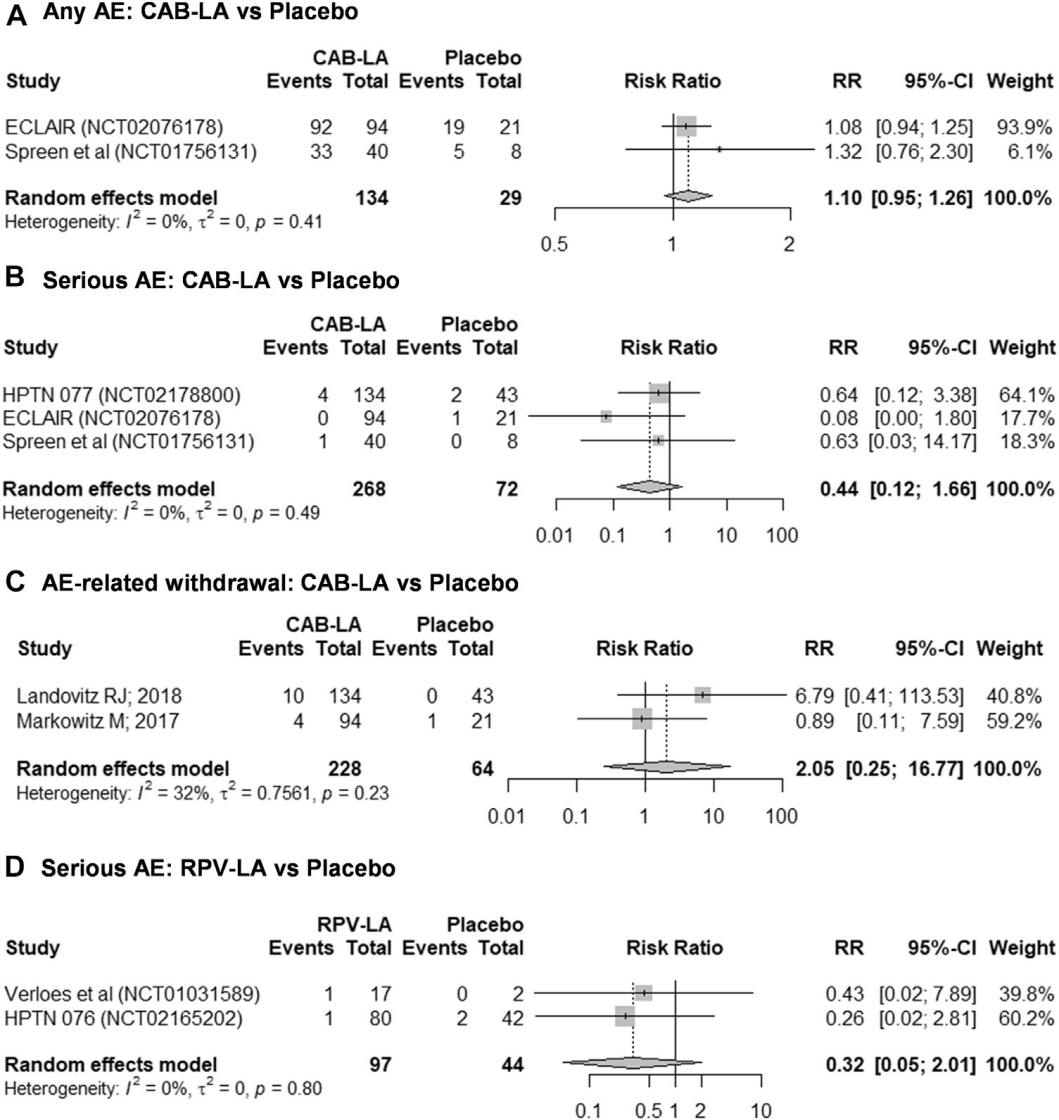
Meta-analyses on safety profiles of **(A–C)** CAB-LA and **(D)** RPV-LA: **(A)** any AE, **(B,D)** serious AE, **(C)** AE-related withdrawal. AE, adverse event; CAB-LA, long-acting cabotegravir; RPV-LA, long-acting rilpivirine.

A total of five studies were included in the meta-analysis (Figures 2A–D). Meta-analysis on the risks of CAB-LA-related AE grade ≥2 was not performed due to substantial heterogeneity (*I* ^2^ > 75%). In addition, we were unable to perform subgroup, sensitivity analyses, and publication bias assessment due to study paucity. Study-specific outcomes on safety profile of LAI PrEP are listed in Supplementary Table S4.

In general, CAB-LA was well-tolerated, with comparable frequencies between intervention and placebo arms of any AE (93.3% [125/134] vs. 82.6% [24/29]; RR 1.10 [95% CI: 0.95–1.26]; *I* ^2^ = 0%; Figure 2A), serious AE (1.9% [5/268] vs. 4.2% [3/72]; RR 0.44 [95% CI: 0.12–1.66]; *I* ^2^ = 0%; Figure 2B), and AE-related withdrawal (6.1% [14/228] vs. 1.6% [1/64]; RR 2.05 [95% CI: 0.25–16.77]; *I* ^2^ = 32%; Figure 2C). However, it is worth noting that the ECLAIR trial reported an increased frequency of AE grade ≥2 (79.8% [75/94] vs. 47.6% [10/21], RR 1.68 [95% CI: 1.06–2.65]) (Markowitz et al., 2017), although the HPTN 077 trial reported a reduced frequency (91.0% [122/134] vs. 88.4% [38/43], RR 1.03 [95% CI: 0.91–1.16]) (Landovitz et al., 2018b).

Likewise, there was no statistical difference in the risk of serious AE between RPV-LA and placebo (2.1% [2/97] vs. 4.5% [2/44], RR 0.32 [95% CI: 0.05–2.01]; *I* ^2^ = 0%; Figure 2D). Nonetheless, given the paucity of studies and the small sample sizes, these findings should be interpreted with caution. Furthermore, we were unable to perform a meta-analysis on the risk of RPV-LA-related AE-related withdrawal as one study reported no events in both arms (Verloes et al., 2015), thus rendering that study ineligible for inclusion in the meta-analysis. Nonetheless, individual studies reported similar frequencies of any AE [82.4% [14/17] vs. 50.0% [1/2], RR 1.65 [95% CI: 0.40–6.70] (Verloes et al., 2015)] and AE grade ≥2 [HPTN 076 trial: 73.8% [31/42] vs. 73.8% [59/80], RR 1.00 [95% CI: 0.80–1.25] (Bekker et al., 2020)].

#### Pharmacokinetic Profiles

**FIGURE 3:**
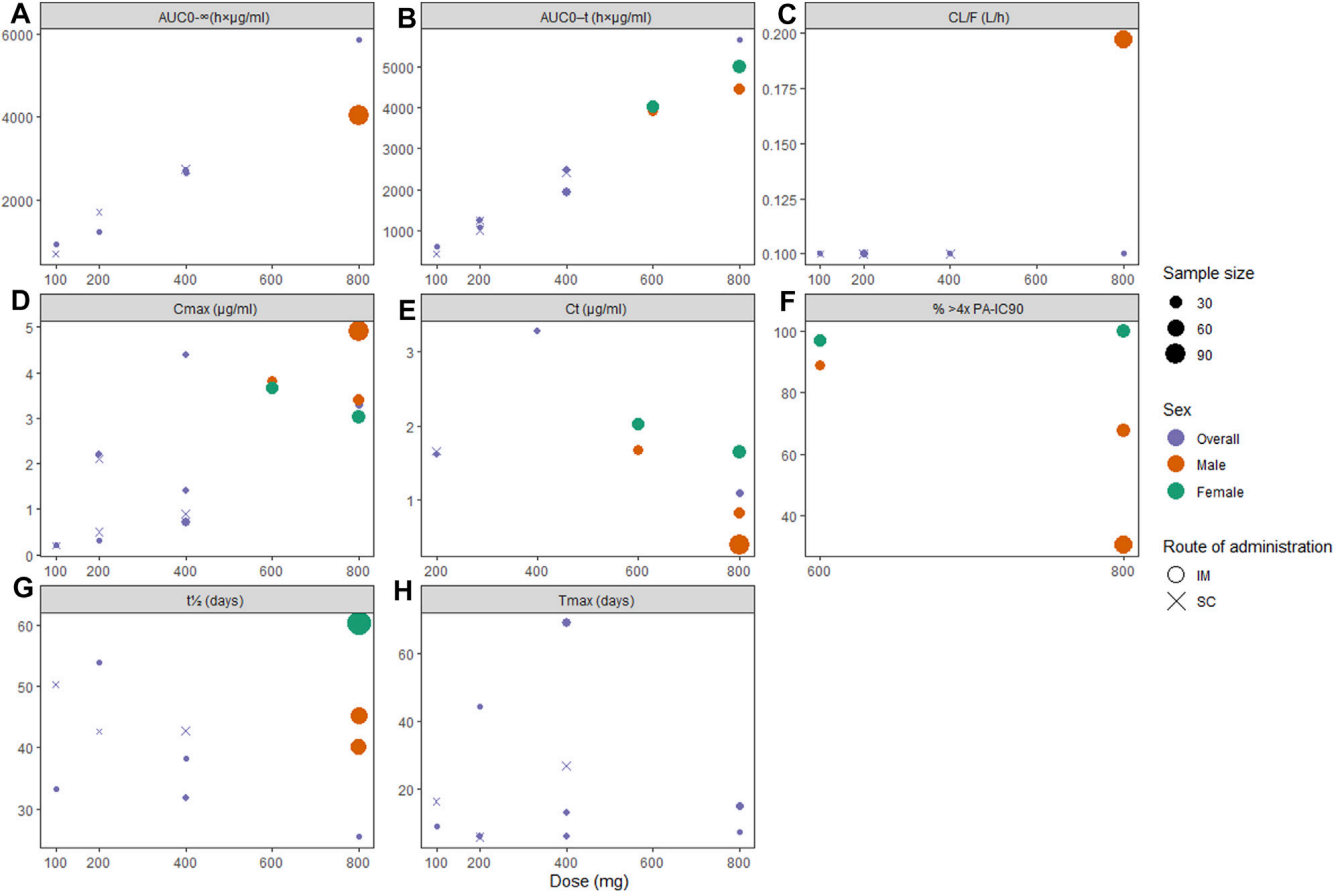
Pharmacokinetic profiles of long-acting cabotegravir following the final injection: **(A)** area under the curve (AUC) from administration time to τ time (AUC_0-τ_), **(B)** AUC from administration time to infinity (AUC_0-ꝏ_), **(C)** apparent clearance (CL/F), **(D)** peak concentration (C_max_), **(E)** concentration through τ time (Cτ), **(F)** proportion of patients with plasma drug concentration >4x PA-IC_90_, **(G)** apparent half-life (t_1/2_), and **(H)** time to peak concentration (T_max_). PA-IC_90_, protein-adjusted inhibitory concentration required for 90% viral inhibition.

**FIGURE 4:**
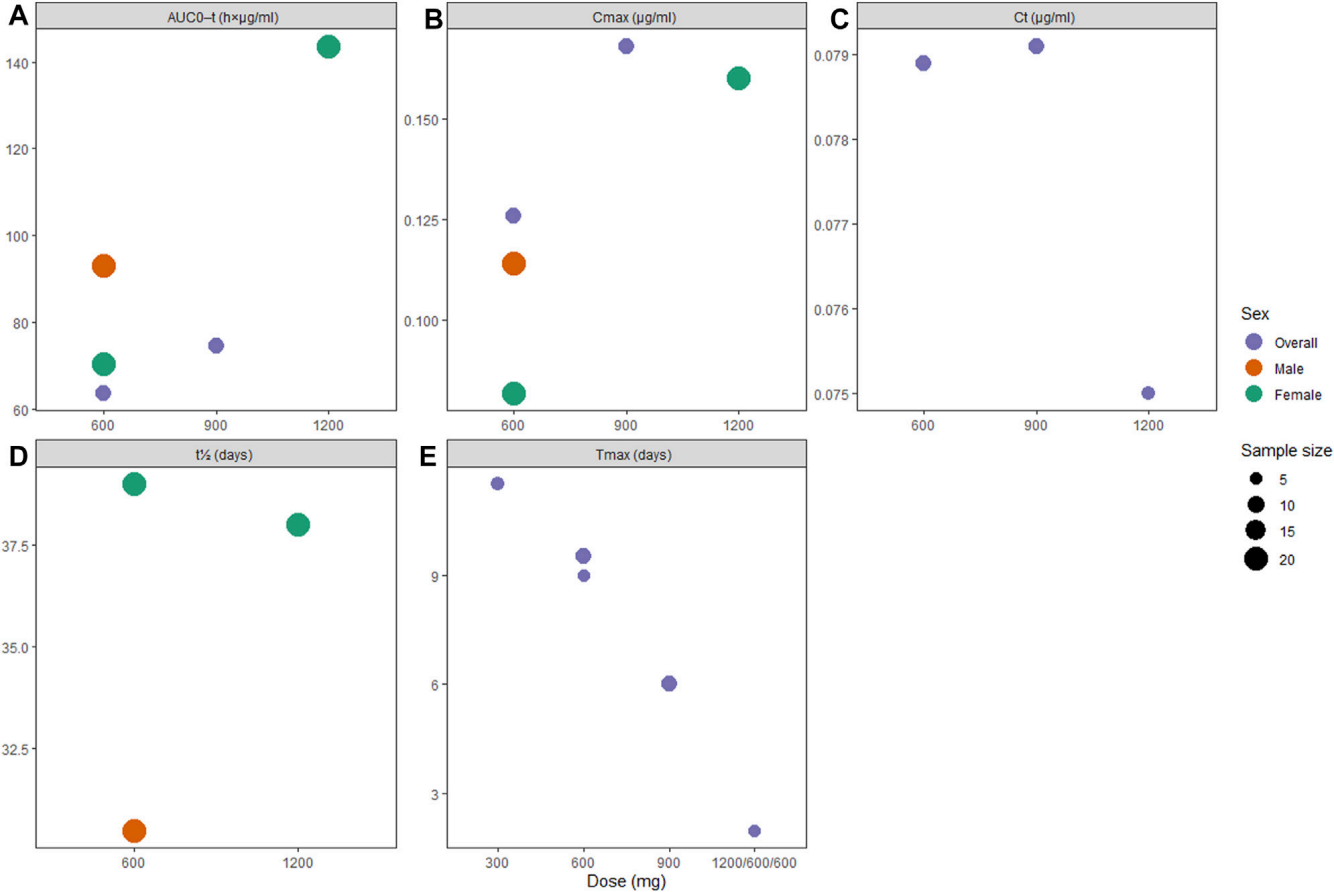
Pharmacokinetic profiles of long-acting rilpivirine following the final injection: **(A)** area under the curve (AUC) from administration time to τ time, **(B)** peak concentration (C_max_), **(C)** concentration through τ time (Cτ), **(D)** apparent half-life (t_1/2_), and **(E)** time to peak concentration (T_max_).

The pharmacokinetic profiles of CAB-LA are summarized in Figures 3A–H, and those of RPV-LA are summarized in Figures 4A–E. For CAB-LA, C_max_, AUC_0-_τ, and AUC_0–∞_ followed dose-response gradients, while CL/F and T_max_ were similar across the dose range (100–800 mg). CAB-LA of 600 mg IM generally yielded similar estimates to those of 800 mg IM; however, there were limited data on the AUC_0–∞_, CL/F, and T_max_ parameters of CAB-LA 600 mg IM. The pharmacokinetic profiles between IM and SC administration of CAB-LA were comparable.

In terms of viral inhibition, five cycles of CAB-LA 600 mg IM Q8W or three cycles of CAB-LA 800 mg IM Q12W yielded satisfactory protection, although more sustainable pharmacokinetics were observed with 600 mg IM Q8W (Figure 3F). Better outcomes were seen in female participants, where 100% in the 800 mg cohort and 96.9% in the 600 mg cohort yielded plasma concentration of >4x PA-IC_90_, as compared to male participants (67.6%) (Landovitz et al., 2018b) and 30.3% (Markowitz et al., 2017) in the 800 mg arm and 88.9% (Landovitz et al., 2018b) in the 600 mg arm). Furthermore, t_1/2_ was also higher among females (60.4 days (Landovitz et al., 2018b)) when compared to males [45.3 (Landovitz et al., 2018b) and 40.0 days (Markowitz et al., 2017)]. Tail-phase analyses revealed that all participants’ plasma drug concentration fell below >4x PA-IC_90_ within 36–52 weeks. In accordance with the findings on t_1/2_, a higher proportion of female participants had a longer duration of plasma concentration >4x PA-IC_90_ (up to 52 weeks, vs. male: up to 36–48 weeks; Supplementary Table S5).

Pharmacokinetic data on RPV-LA showed subtle dose-dependent gradient in terms of AUC_0-_τ and C_max_ (Figures 4A,B), while the T_max_ parameter showed inverted dose-dependence where increasing dose required less time to reach peak concentrations (Figure 4E). Similar to our findings on CAB-LA, t_1/2_ in RPV-LA was longer in female participants (38.0 [1,200 mg IM] and 39.0 [600 mg IM] vs. 30.5 days [600 mg IM]; Supplementary Table S6). However, viral inhibition was only satisfactory for 1,200 mg IM (Jackson et al., 2014; Verloes et al., 2015; Bekker et al., 2020), while other single doses failed to consistently reach >4x PA-IC_90_ (50 ng/ml) (Jackson et al., 2014). Nonetheless, following a loading dose of 1,200 mg IM, both 600 and 900 mg IM successfully maintained plasma concentration of >4x PA-IC_90_ until about 12 weeks (Spreen et al., 2014b), although it is worth noting that Verloes et al. reported that follow-up injections of 600 mg IM Q4W may be suboptimal as some of the patients’ plasma drug concentration fell below 4x PA-IC_90_ (Verloes et al., 2015).

## Discussion

Long-acting (LA) injectable HIV-1 pre-exposure prophylaxis (PrEP) are among the pinnacles of HIV PrEP as they may potentially provide long-term protection by creating a depot-controlled nanosuspension (Nyaku et al., 2017). To our knowledge, this is the first systematic review that investigated the safety and pharmacokinetic profiles of LAI PrEP, notably CAB-LA and RPV-LA, confirming that LAI PrEP of RPV-LA and CAB-LA have satisfactory safety and pharmacokinetic profiles. Although data on RPV-LA’s safety profiles were limited, preliminary evidence favored the safety and tolerability of these drugs, which may be further ascertained by forthcoming studies [HPTN 083 [NCT02720094] (HIV Prevention Trials Network, 2020a) and HPTN 084 [NCT03164564] (HIV Prevention Trials Network, 2020b)]. In addition, although the ECLAIR trial suggested that CAB-LA may be associated with an increased risk of AE grade ≥2, these were mostly related to injection-site reactions, which were manageable and not life-threatening (Markowitz et al., 2017) In the study by Spreen et al., most participants rated the tolerability of LAI PrEP as ≥4.5 out of five, further supporting high acceptability (Spreen et al., 2014b).

Although both 800 mg IM Q12W and 600 mg IM Q8W of CAB-LA successfully reached a plasma drug concentration of >4x PA-IC_90_, 600 mg IM Q8W may be preferred as it consistently maintained the desired plasma concentration, unlike 800 mg IM Q12W (Markowitz et al., 2017; Landovitz et al., 2018b). This is further supported by the fact that pharmacokinetics profiles of these two dosing regimens were similar, and that the number of injection site reactions and adverse event-related drug discontinuation were slightly higher in the 800 mg IM Q12W arm (Landovitz et al., 2018b).

Striking differences were observed in CAB-LA plasma concentrations between males and females, suggesting high variability in absorption. A potential explanation is the disparity in muscle size and fat distribution, in addition to host genetics (Landovitz R. J. et al., 2020), which concurred with the longer half-life of CAB-LA observed in female volunteers and participants with higher body mass index (BMI). This finding suggests that careful selection of injection sites with regards to body fat distribution is imperative (Landovitz R. J. et al., 2020). To date, IM injection site for CAB-LA is only recommended in the gluteal area (Landovitz et al., 2018b). Thus, further study is needed to explore additional injection site options (Landovitz et al., 2018b). Although route-wise comparison revealed comparable pharmacokinetic profiles, IM injections are preferred due to fewer injection site reactions (Spreen et al., 2014a; Spreen et al., 2014b). This may render CAB-LA IM the preferred drug especially considering that adverse drug reactions were among the most common reasons leading to poor adherence (Leporini et al., 2014).

One potential limitation of CAB-LA is the possibility of prolonged drug decay with suboptimal protection following cessation, which may create a period of susceptibility to HIV infection and selection of drug-resistant HIV strains (Landovitz R. J. et al., 2020). Although the current evidence did not show any emergence of resistance mutations (Markowitz et al., 2017; Landovitz et al., 2018b), further phase III trials and post-approval data are required to ascertain these findings. As compared to the currently approved daily oral PrEP regimen (i.e., tenofovir disoproxil fumarate/emtricitabine), the likelihood of selecting drug-resistant variants may be either lower due to the higher resistance barrier of CAB-LA or higher due to the use of a single ARV drug (Landovitz R. J. et al., 2020) Nonetheless, this indicates that even though LAI CAB may solve barriers to adherence with oral PrEP (Nyaku et al., 2017), patients’ adherence will continue to play an important role in the effectiveness of LAI CAB. This emphasizes that LAI PrEP has to be complemented by adherence support strategies to maximize the potentials of these agents, including through maintenance support, cognitive strategies, and recurring reminders (Mayer et al., 2017; Grov et al., 2019).

Similar to the pharmacokinetic profiles of CAB-LA, RPV-LA also had favorable pharmacokinetic parameters. According to our findings, RPV-LA doses of 1,200 mg IM may sufficiently achieve the desired plasma concentration (Spreen et al., 2014b; Bekker et al., 2020). Furthermore, maintenance doses of 1,200 mg Q8W or 900 mg may be preferable over 600 mg (Spreen et al., 2014b; Bekker et al., 2020), especially considering that 600 mg IM injection had failed to consistently reach the needed concentration (Spreen et al., 2014b; Verloes et al., 2015). Nevertheless, it should be noted that only 29.7% of participants had satisfactory plasma concentration following a single 1,200 mg IM RPV-LA injection, while the proportion reached >80% only after the third injection (Bekker et al., 2020), thus necessitating concomitant preventive strategies during the initial phase to ensure adequate protection against high-risk HIV-1 exposure.

In addition to the tolerability and dosing of the regimens, the optimal timing to initiate LAI PrEP is important. To date, the time needed to obtain full protection against HIV infection following the initial PrEP injection remains unknown. For CAB-LA, Landovitz et al. set a target median C_τ_ of 1.35 μg/ml, which predicts a plasma C_τ_ of ≥4×PA-IC_90_ in at least 80% participants and ≥PA-IC_90_ in at least 95% participants (Landovitz et al., 2018b). Based on this target, a dosing regimen of 600 mg IM Q8W (preceded by an initial Q4W injection) may reach an optimum protection after about one month into the cycle (Landovitz et al., 2018b), while the other dosing strategies failed to sustain the desired concentration (Spreen et al., 2014a; Spreen et al., 2014b; Markowitz et al., 2017; Landovitz et al., 2018b). Previous reports showed that drug tissue concentrations were remarkably lower than the corresponding plasma concentrations, which may imply that the time needed to achieve adequate protection in cervicovaginal and rectal tissues may be longer (Spreen et al., 2014a). This warrants further studies of the pharmacokinetics of 600 mg IM Q8W in cervicovaginal and rectal tissues.

Scarce data were available for RPV-LA as the target PA-IC_90_ value was arbitrary without any known relationship between plasma concentration and preventive efficacy (Bekker et al., 2020). While the current findings indicated that RPV-LA 1200 mg IM Q8W may achieve plasma concentrations of ≥4xPA-IC_90_ in at least 80% participants in about four months after the initial injection (Bekker et al., 2020), exploration of other dosing strategies is required to obtain the most optimal prophylactic regimen strategy to achieve the desired pharmacokinetics more readily. The current RPV-LA formulation requires cold-chain storage, which may be impractical in low-resource settings (Bekker et al., 2020), warranting further optimization of the current RPV-LA formulations. Given the long-acting nature of the products (Landovitz et al., 2018b), careful patient selection is required to ensure the maximal efficacy of the drugs while simultaneously preventing selection of drug-resistant HIV strains. In this case, patients with a continued risk of HIV infection may benefit most from the LAI PrEP.

Altogether, these findings support the potential utility of LAI PrEP in preventing HIV transmission. Previous reports have suggested higher rates of acceptability and preference of LAI PrEP, compared to oral PrEP (Meyers et al., 2014; Murray et al., 2018) or vaginal ring or gel (Bekker et al., 2020). LAI PrEP may also have important additional advantages over oral PrEP as it may minimize drug-drug interactions by bypassing food effects and first-pass metabolism (Verloes et al., 2015). Ultimately, the long-awaited efficacy results from ongoing phase 3 trials may provide indispensable information on the preventive utility of these agents (National Institute of Allergy and Infectious Diseases, 2016; National Institute of Allergy and Infectious Diseases, 2017b). The interim data of HTPN083 and HTPN084 presented at the AIDS conference in July 2020 showed that CAB-LA had superior preventive efficacy over oral PrEP (HPTN 083: −66% in homosexual cisgender men and transgender women [hazard ratio 0.34, 95% CI: 0.18–0.62]; HPTN 084: −89% in cisgender women) and that CAB-LA were well-tolerated and had similar safety profiles to oral PrEP (Landovitz R. et al., 2020; HIV Prevention Trials Network, 2020c).

There were some study limitations. Studies reported different measures of dispersion, thus precluding pooled analyses of the pharmacokinetic parameters. Because of scarce data, we could not perform further subgroup analyses and assess potential sources of heterogeneity; further trial data are needed to confirm our findings. Nonetheless, the predominant studies had low risk of bias which strengthened the validity of our findings.

In conclusion, our findings add to the growing body of evidence supporting the tolerability and favorable pharmacokinetics of LAI PrEP, notably CAB-LA and RPV-LA. Both CAB-LA and RPV-LA were well-tolerated with similar safety profiles to placebo. CAB-LA 600 mg IM Q8W yielded satisfactory pharmacokinetics and viral inhibitory activity, and the same were observed with RPV-LA 1200 mg IM Q8W. Further research is required to confirm the safety and pharmacokinetic profiles of RPV-LA as the current evidence is limited.

## Data Availability

The original contributions presented in the study are included in the article/Supplementary Material, further inquiries can be directed to the corresponding author.

## Abbreviations

%CV, Coefficient of variability; AE, Adverse events; AIDS, Acquired immunodeficiency syndrome; ARV, Antiretroviral; AUC_0-τ_, Area under the plasma drug concentration through τ time; AUC_0–∞_, Area under the plasma drug concentration from time 0 to infinity; BMI, Body mass index; C_τ_, Plasma drug concentration through τ time; C_max,_ Peak plasma drug concentration; CAB-LA, Long-acting injectable cabotegravir; CENTRAL, Cochrane Controlled Register of Trials; CL/F, Apparent clearance; CI, Confidence interval; CINAHL, Cumulative Index to Nursing and Allied Health Literature; HIV, Human immunodeficiency virus; HIV-1, Human immunodeficiency virus type 1; IM, Intramuscular; LAI, Long-acting injectable; PA-IC_90,_ 90% protein-adjusted inhibitory concentration; PrEP, Pre-exposure prophylaxis; PRISMA, Preferred Reporting Items for Systematic Reviews and Meta-Analyses; RPV-LA, Long-acting injectable rilpivirine; RR, Risk ratio; SC, Subcutaneous; T_max_, Time to peak plasma drug concentration; t_1/2_, Apparent half life.
