# Supplementary material for: Safety and Pharmacokinetic Profiles of Long-Acting Injectable Antiretroviral Drugs for HIV-1 Pre-Exposure Prophylaxis: A Systematic Review and Meta-analysis of Randomized Trials
Source: Front Pharmacol. 2021 Jul 7;12:664875. doi: 10.3389/fphar.2021.664875 (PMC8299834; doi:10.3389/fphar.2021.664875)
Supplement: Supplementary file 1 [file DataSheet1.pdf]

frontiers

Supplementary Material

Safety and pharmacokinetic profiles of long-acting injectable antiretroviral drugs for HIV-1 pre-exposure prophylaxis: a systematic review and meta-analysis of randomized trials

Gilbert Lazarus[1], Vincent Kharisma Wangsaputra[1,†], Christianto[1,†], Melva Louisa[2*], Vivian Soetikno[2], Raph L. Hamers[1,3,4]

[1]Faculty of Medicine, Universitas Indonesia, Jakarta, Indonesia

[2]Department of Pharmacology and Therapeutics, Faculty of Medicine, Universitas Indonesia, Jakarta, Indonesia.

[3]Eijkman-Oxford Clinical Research Unit, Universitas Indonesia, Jakarta, Indonesia

[4]Centre for Tropical Medicine and Global Health, Nuffield Department of Medicine, University of Oxford, Oxford, UK

*Corresponding author: melva.louisa@gmail.com

[†]These authors contributed equally

Table and Contents

**Supplementary Material**	Page
**Methods**	3
Table S1. Deviations from the protocol	3
Table S2. Search strategy	3
Definitions	5
**Results**	7
Figure S1. Study-specific results of risk of bias assessment	7
Figure S2. Summary of risk of bias assessments	7

Table S3. Risk-of-bias assessments of RCTs using Cochrane Risk-of-Bias Tool for Randomized Trials ver. 2.0. (Cochrane RoB 2)	8
Table S4. Safety profiles of long-acting injectable pre-exposure prophylaxis	12
Table S5. Pharmacokinetic profiles of long-acting injectable cabotegravir after the final injection	13
Table S6. Pharmacokinetic profiles of long-acting injectable rilpivirine after the final injection	18
**References**	21

METHODS

Table S1. Deviations from the protocol (PROSPERO CRD42020154772(Lazarus and Christianto))

No	Deviations	Explanations
1	Additional safety outcomes	Initially, we only aimed to investigate the risk of developing AE grade ≥2 to represent the safety profiles of LAI PrEP. We deemed that AE of grade <2 was not clinically significant to determine the safety profiles of LAI PrEP as these AEs are usually self-limited and/or relieved spontaneously.<br><br>However, upon data abstraction, we discovered that AE grade ≥2 was poorly reported in half of the included studies. In order to obtain a better representation on the safety profiles of LAI PrEP, we decided to add additional outcomes including the number of patients: (1) experiencing any AE, (2) experiencing serious AE, and (3) withdrawing treatment due to AE.
2	Additions to meta-analysis method	Initially, we aimed to perform meta-analysis using fixed-effects method. Upon data abstraction, we discovered remarkable variability in the dosing regimens and intervals as well as follow-up periods, which we deem may potentially introduce heterogeneity to our findings. Hence, we decided to perform meta-analysis using random-effects model<br><br>Furthermore, given the fact that we were unable to perform subgroup and sensitivity analyses to explore potential sources of heterogeneity due to study paucity, we only performed meta-analysis when heterogeneity does not pose significant threats to the validity of our findings (I^2<75% and p-value >0.01). Although these cut-offs were arbitrary, these values indicate considerable heterogeneity to the model, thus implying potentially misleading estimates if pooled.(Higgins et al., 2019)

Table S2. Search strategy

Database	Keywords
PubMed (83)	((("hiv"[MeSH Terms]) OR ("hiv infections"[MeSH Terms]) OR ("acquired immunodeficiency syndrome"[MeSH Terms]) OR ("human immunodeficiency virus proteins"[MeSH Terms])) AND (("rilpivirine"[MeSH Terms]) OR ("integrase inhibitors"[MeSH Terms]) OR ("reverse transcriptase inhibitors"[MeSH Terms]) OR ("protease inhibitors"[MeSH Terms]) OR ("anti-retroviral agents"[MeSH Terms]) OR ("cabotegravir"[All Fields]) OR

	("TMC278"[All Fields]) OR ("GSK1265744"[All Fields])) AND (("Primary Prevention"[MeSH Terms]) OR ("pre-exposure prophylaxis"[MeSH Terms]) OR ("hiv seronegativity"[MeSH Terms]) OR ("HIV-uninfected"[All Fields])) AND (("long-acting injectable"[All Fields]) OR ("parenteral"[All Fields]) OR ("injections"[MeSH Terms]) OR ("injectable"[All Fields]) OR ("sustained release"[All Fields])))
CENTRAL (20)	#1 MeSH descriptor: [HIV] explode all trees<br>#2 MeSH descriptor: [HIV Infections] explode all trees<br>#3 MeSH descriptor: [Acquired Immunodeficiency Syndrome] explode all trees<br>#4 MeSH descriptor: [Rilpivirine] explode all trees<br>#5 MeSH descriptor: [Integrase Inhibitors] explode all trees<br>#6 MeSH descriptor: [Anti-Retroviral Agents] explode all tree<br>#7 MeSH descriptor: [Reverse Transcriptase Inhibitors] explode all trees<br>#8 MeSH descriptor: [Pre-Exposure Prophylaxis] explode all trees<br>#9 MeSH descriptor: [Primary Prevention] explode all trees<br>#10 MeSH descriptor: [HIV Seronegativity] explode all trees<br>#11 MeSH descriptor: [Injections] explode all trees<br>#12 {OR #1-#3}<br>#13 {OR #4-#7} OR "cabotegravir" OR "TMC278" OR "GSK1265744"<br>#14 {OR #8-#10} OR "HIV-uninfected"<br>#15 #11 OR "long-acting injectable" OR "parenteral" OR "injectable" OR "sustained release"<br>#16 {AND #12-#16}
Scopus (642)	(((TITLE-ABS-KEY("long-acting injectable")) OR (TITLE-ABS-KEY("parenteral")) OR (TITLE-ABS-KEY("sustained release")) OR (TITLE-ABS-KEY(“injections”))) AND ((TITLE-ABS-KEY("pre-exposure prophylaxis") OR TITLE-ABS-KEY("prevention"))) AND ((TITLE-ABS-KEY("cabotegravir")) OR (TITLE-ABS-KEY("rilpivirine")) OR (TITLE-ABS-KEY("GSK1265744")) OR (TITLE-ABS-KEY("TMC278")) OR (TITLE-ABS-KEY("anti*retroviral”)) OR (TITLE-ABS-KEY("integrase inhibitor")) OR (TITLE-ABS-KEY("protease inhibitor")) OR (TITLE-ABS-KEY("reverse transcriptase inhibitor"))))
CINAHL (34), EBSCO MEDLINE (71)	S1 (MH "Human Immunodeficiency Virus+") OR (MH "HIV Infections+") OR "HIV" OR (MH "Acquired Immunodeficiency Syndrome")<br>S2 (MH "Anti-HIV Agents+") OR (MH "HIV Entry and Fusion Inhibitors+") OR (MH "HIV Integrase Inhibitors+") OR (MH "HIV Protease Inhibitors+") OR (MH "Anti-Retroviral Agents+") OR (MH "Rilpivirine") OR (MH "Nucleoside Reverse Transcriptase Inhibitors+") OR (MH "Non-Nucleoside Reverse Transcriptase Inhibitors+") OR ("cabotegravir") OR ("TMC278") OR ("GSK1265744")

	S3 ((MH "Preventive Health Care+") OR (MH "Pre-Exposure Prophylaxis") OR (MH "HIV Seronegativity") OR ("primary prevention") OR ("HIV-uninfected")<br>S4 (MH "Injections+") OR ("long-acting injectable") OR ("parenteral") OR ("injectable") OR ("sustained release")<br>S5 S1 AND S2 AND S3 AND S4
Google Scholar (54)	(("cabotegravir" OR "rilpivirine" OR "GSK1265744" OR "TMC278" OR "antiretroviral") AND ("HIV" OR "AIDS" OR "human immunodeficiency virus") AND ("long-acting injectable" OR "parenteral" OR "sustained release" OR "injections"))
ProQuest (1369)	((("hiv") OR ("hiv infections") OR ("acquired immunodeficiency syndrome") OR ("human immunodeficiency virus proteins")) AND (("rilpivirine") OR ("integrase inhibitors") OR ("reverse transcriptase inhibitors") OR ("protease inhibitors") OR ("Anti-Retroviral Agents") OR ("cabotegravir") OR ("TMC278") OR ("GSK1265744")) AND (("Primary Prevention") OR ("Pre-exposure Prophy-laxis") OR ("HIV-uninfected")) AND (("long-acting injectable") OR ("parenteral") OR ("injections") OR ("injectable") OR ("sustained release")))
Clinicaltrials.gov (17)	Condition or disease: HIV<br>Other terms: (Rilpivirine OR Cabotegravir) AND (Injectable OR Injection OR Parenteral) AND (Prevention OR Prophylaxis)

Definitions

Safety

Whenever possible, safety outcomes were defined according to the corrected version 2.1 of Division of AIDS (DAIDS) Table for Grading the Severity of Adult and Pediatric Adverse Events(National Institute of Allergy and Infectious Diseases, 2017). Adverse event (AE) was defined as any untoward event (i.e. sign, symptoms, disease) temporally associated with medical procedures or treatments, regardless of its causalities. According to the grading criteria(National Institute of Allergy and Infectious Diseases, 2017), adverse events grade ≥2 encompass moderate-or-higher AE severity, including moderate (grade 2), severe (grade 3), life-threatening events (grade 4), and events resulting in death (grade 5). On the other hand, serious AE was defined as AE resulting in death, life-threatening situations, significant impairments, birth defects or requiring immediate intervention, inpatient care, or prolongation of hospitalization.(National Institute of Allergy and Infectious Diseases, 2017)

Pharmacokinetics

In this study, pharmacokinetics profile was investigated using peak concentrations (C_{max}), time to peak concentrations (T_{max}), area under the plasma drug concentration under through τ time ($AUC_{0\text{-}\tau}$), AUC from time 0 (administration time) to infinity ($AUC_{0-\infty}$), apparent half-life ($t_{1/2}$), apparent clearance (CL/F), and proportion of patients with plasma drug concentration of >4x 90% protein-adjusted inhibitory concentration (PA-IC_{90}). C_{max}, T_{max}, $t_{1/2}$, AUC, and CL/F illustrate the kinetic profiles of the drug, while PA-IC_{90} provides information on the viral inhibition potentials of the drug. AUC is a parameter measuring the amount of drug entering the systemic circulation and requiring clearance. As LAI PrEP is usually administered with fixed time intervals, we collected both AUC from administration time to infinity ($AUC_{0-\infty}$) and AUC from administration time to dosing interval ($AUC_{0\text{-}\tau}$).(Urso et al., 2002) $AUC_{0\text{-}\tau}$ is generally expected to lie closely to $AUC_{0-\infty}$ when clearance rate remains constant for a drug, regardless of its administered concentrations. Furthermore, $AUC_{0-\infty}$ provides additional useful information by allowing the calculation of CL/F and $t_{1/2}$. In this review, apparent half-life was utilized instead of terminal half-life considering that the drug was injected in controlled-release preparations, indicating that the declining drug plasma concentration was not solely due to elimination. Four half-lives were generally required for a therapeutic effect of a drug to be deemed negligible.(Benet, 1984)

On the other hand, we defined plasma drug concentration of >4x PA-IC_{90} as providing satisfactory protection based on preliminary data. As stated in the HPTN 077 trial, no HIV transmissions occurred when the plasma drug concentration remained above 4x PA-IC_{90}, indicating sufficient antiviral protection. Although most samples were also protected even when plasma concentrations were only slightly above PA-IC_{90}, these borderline concentrations still pose imminent risks of HIV transmission.(Landovitz et al., 2018b)

As pharmacokinetic parameters are usually skewed, we chose geometric mean along with its 95% confidence interval (CI) as the common effect measure. However, when only between-person coefficient of variability (%CV) was reported as an estimate of standard error, we decided to extract the data as is as we were unable to obtain a reliable approximation between %CV and 95% CI. %CV was defined as percent of relative standard deviation (dispersion) of the estimate, and a high %CV value was deemed unfavorable as it indicates uncertainty in the variability of key pharmacokinetic parameters.(Grabowski et al., 2014) When only 90% CI was reported, we converted the parameter to 95% CI as per guideline(Higgins et al., 2019).

RESULTS

Figure S1. Study-specific results of risk of bias assessment

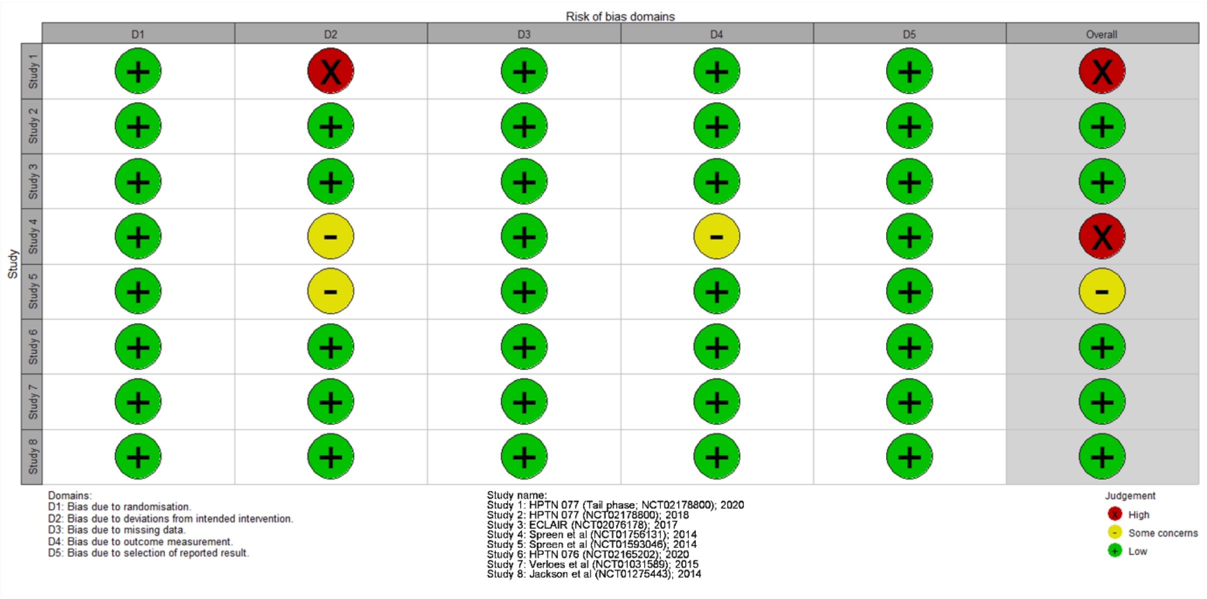

Figure S2. Summary of risk of bias assessments

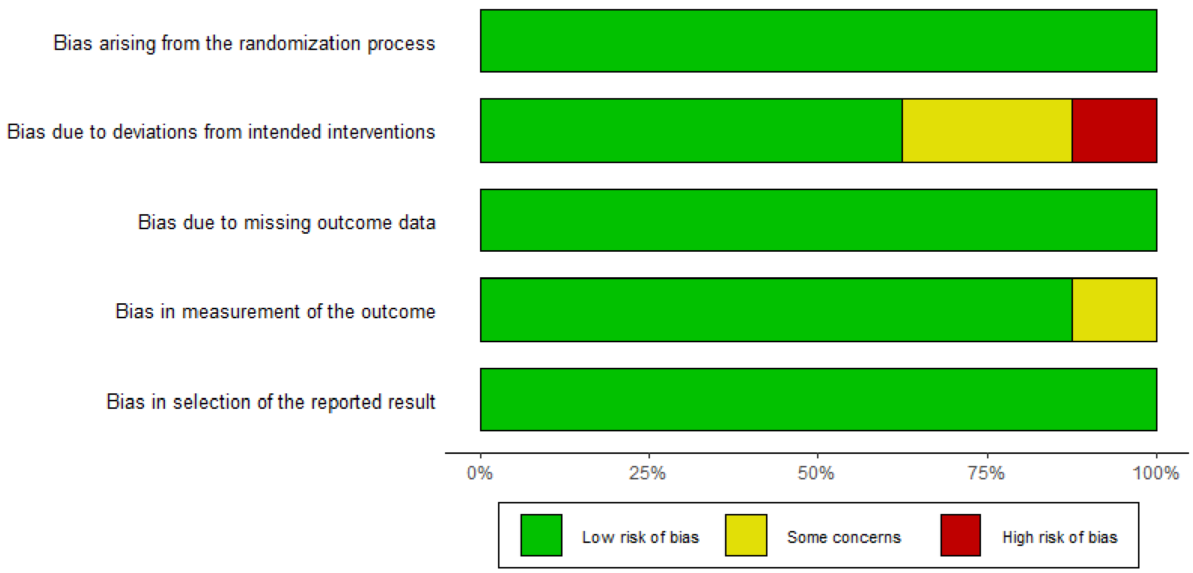

Table S3. Risk-of-bias assessments of RCTs using Cochrane Risk-of-Bias Tool for Randomized Trials ver. 2.0. (Cochrane RoB 2)(Sterne et al., 2019)

**Bias domain**	**Signalling questions**	**Response options**	**Description/Support for judgement**							
			**HPTN 077 (Tail-phase); 2020**	**HPTN 077; 2018**	**ECLAIR; 2017**	**Spreen et al (NCT01756131); 2014**	**Spreen et al (NCT01593046); 2014**	**HPTN 076; 2020**	**Verloes et al (NCT01031589); 2015**	**Jackson et al (NCT01275443); 2014**
Bias arising from the randomization process	1.1 Was the allocation sequence random?	Y / PY / PN / N / NI	Y	Y	Y	Y	Y	Y	PY	Y
	1.2 Was the allocation sequence concealed until participants were enrolled and assigned to interventions?	Y / PY / PN / N / NI	Y	Y	Y	NI	PY	Y	PY	Y
	1.3 Did baseline differences between intervention groups suggest a problem with the randomization process?	Y / PY / PN / N / NI	PN	N	N	NI	N	PN	N	PN
	**Risk of bias judgement**	**- / + / ?**	**-**	**-**	**-**	**-**	**-**	**-**	**-**	**-**
Domain 2: Risk of bias due to deviations from the intended	2.1. Were participants aware of their assigned intervention during the trial?	Y / PY / PN / N / NI	N	N	N	Y	Y	N	PN	Y
	2.2. Were carers and people delivering the interventions aware of participants' assigned intervention during the trial?	Y / PY / PN / N / NI	PN	PN	N	Y	Y	Y	PN	Y
	2.3. If Y/PY/NI to 2.1 or 2.2: Were there deviations from the intended intervention that arose because of the experimental context?	NA / Y / PY / PN / N / NI	NA	NA	NA	NI	NI	PN	NA	PN

interventions (*effect of assignment to intervention*)	2.4. If Y/PY to 2.3: Were these deviations from intended intervention balanced between groups?	NA / Y / PY / PN / N / NI	NA	NA	NA	NA	NA	NA	NA	NA
	2.5 If N/PN/NI to 2.4: Were these deviations likely to have affected the outcome?	NA / Y / PY / PN / N / NI	NA	NA	NA	NA	NA	NA	NA	NA
	2.6 Was an appropriate analysis used to estimate the effect of assignment to intervention?	Y / PY / PN / N / NI	PY	Y	PY	Y	Y	Y	Y	Y
	2.7 If N/PN/NI to 2.6: Was there potential for a substantial impact (on the result) of the failure to analyse participants in the group to which they were randomized?	NA / Y / PY / PN / N / NI	NA	NA	NA	NA	NA	NA	NA	NA
	**Risk of bias judgement**	**- / + / ?**	**-**	**-**	**-**	**?**	**?**	**-**	**-**	**-**
Domain 2: Risk of bias due to deviations from the intended interventions (*effect of adhering to*	2.1. Were participants aware of their assigned intervention during the trial?	Y / PY / PN / N / NI	N	N	N	Y	Y	N	PN	Y
	2.2. Were carers and people delivering the interventions aware of participants' assigned intervention during the trial?	Y / PY / PN / N / NI	PN	PN	N	Y	Y	N	PN	Y
	2.3. If Y/PY/NI to 2.1 or 2.2: Were important co-interventions balanced across intervention groups?	NA / Y / PY / PN / N / NI	NA	NA	NA	Y	Y	NA	NA	PY
	2.4. Were there failures in implementing the intervention that could have affected the outcome?	Y / PY / PN / N / NI	PY	PN	N	PY	NI	NI	N	N
	2.5. Did study participants adhere to the assigned intervention regimen?	Y / PY / PN / N / NI	PN	PY	Y	PN	PN	PY	Y	PY

intervention)	2.6. If N/PN/NI to 2.3 or 2.5 or Y/PY/NI to 2.4: Was an appropriate analysis used to estimate the effect of adhering to the intervention?	NA / Y / PY / PN / N / NI	PN	NA	NA	Y	Y	NA	NA	NA
	**Risk of bias judgement**	**- / + / ?**	+	-	-	?	?	-	-	-
Bias due to missing outcome data	3.1 Were data for this outcome available for all, or nearly all, participants randomized?	Y / PY / PN / N / NI	PN	PY	Y	PN	PN	PY	PY	Y
	3.2 If N/PN/NI to 3.1: Is there evidence that the result was not biased by missing outcome data?	NA / Y / PY / PN / N	PY	NA	NA	PY	Y	NA	NA	NA
	3.3 If N/PN to 3.2: Could missingness in the outcome depend on its true value?	NA / Y / PY / PN / N / NI	NA	NA	NA	NA	NA	NA	NA	NA
	3.4 If Y/PY/NI to 3.3: Is it likely that missingness in the outcome depended on its true value?	NA / Y / PY / PN / N / NI	NA	NA	NA	NA	NA	NA	NA	NA
	**Risk of bias judgement**	**- / + / ?**	-	-	-	-	-	-	-	-
Bias in measurement of the outcome	4.1 Was the method of measuring the outcome inappropriate?	Y / PY / PN / N / NI	N	N	N	N	N	N	N	N
	4.2 Could measurement or ascertainment of the outcome have differed between intervention groups?	Y / PY / PN / N / NI	N	N	NI	NI	PN	PN	PN	PN
	4.3 If N/PN/NI to 4.1 and 4.2: Were outcome assessors aware of the intervention received by study participants?	Y / PY / PN / N / NI	PN	N	N	Y	Y	N	PN	PN
	4.4 If Y/PY/NI to 4.3: Could assessment of the outcome have been influenced by knowledge of intervention received?	NA / Y / PY / PN / N / NI	NA	NA	NA	NI	PN	NA	NA	NA

	4.5 If Y/PY/NI to 4.4: Is it likely that assessment of the outcome was influenced by knowledge of intervention received?	NA / Y / PY / PN / N / NI	NA	NA	NA	PN	NA	NA	NA	NA
	**Risk of bias judgement**	**- / + / ?**	**-**	**-**	**-**	**?**	**-**	**-**	**-**	**-**
Bias in selection of the reported result	5.1 Were the data that produced this result analysed in accordance with a pre-specified analysis plan that was finalized before unblinded outcome data were available for analysis?	Y / PY / PN / N / NI	PY	Y	Y	PY	PY	PY	PY	Y
	Is the numerical result being assessed likely to have been selected, on the basis of the results, from...									
	5.2. ... multiple outcome measurements (e.g. scales, definitions, time points) within the outcome domain?	Y / PY / PN / N / NI	N	N	N	N	N	PN	PN	PN
	5.3 ... multiple analyses of the data?	Y / PY / PN / N / NI	N	N	N	N	N	N	N	N
	**Risk of bias judgement**	**- / + / ?**	**-**	**-**	**-**	**-**	**-**	**-**	**-**	**-**
**Overall bias**	**Risk of bias judgement**	**- / + / ?**	+	-	-	+	?	-	-	-

*Give information separately for exposed and unexposed groups; (-), low risk of bias; (?), some concerns; (+), high risk of bias.

Table S4. Safety profiles of long-acting injectable pre-exposure prophylaxes[a]

Author/Trial name (Trial ID); Year	AE grade ≥2		Any AE		Serious AE		AE-related withdrawal	
	I	C	I	C	I	C	I	C
**Cabotegravir LA**								
HPTN 077 (Tail phase; NCT02178800)(Landovitz et al., 2018a); 2020 (Incidence per 100 person-years [95% CI])	Tail-phase: 214.9 (180.3-256.1) Injection-phase: 750.0 (662.3-849.2)	NR	NR	NR	NR	NR	NR	NR
HPTN 077 (NCT02178800)(Landovitz et al., 2018b); 2018	122/134 (91.0)	38/43 (88.4)	NR	NR	4/134 (3.0)	2/43 (4.7)	10/134 (7.5)	0/43 (0.0)
ECLAIR (NCT02076178)(Markowitz et al., 2017); 2017	60/94 (63.8)	10/21 (47.6)	92/94 (97.9)	19/21 (90.5)	0/94 (0.0)	1/21 (4.8)	4/94 (4.3)	1/21 (4.8)
Spreen et al (NCT01756131)(Spreen et al., 2014a); 2014	NR	NR	IM: 33/40 (82.5) SC: 17/18 (94.4)	IM: 5/8 (62.5) SC: 5/6 (83.3)	IM: 1/40 (2.5) SC: 1/18 (5.6)	IM: 0/8 (0.0) SC: 0/6 (0.0)	IM: 0/40 (0.0) SC: 0/18 (0.0)	IM: 0/8 (0.0) SC: 0/6 (0.0)
Spreen et al (NCT01593046)(Spreen et al., 2014b); 2014	NR	NA	NR	NA	NR	NA	NR	NA
**Rilpivirine LA**								
HPTN 076 (NCT02165202)(Bekker et al., 2020); 2020	59/80 (73.8)	31/42 (73.8)	NR	NR	1/80 (1.3)	2/42 (4.8)	6/80 (7.5)	2/42 (4.7)
Verloes et al (NCT01031589)(Verloes et al., 2015); 2015	NR	NR	14/17 (82.4)	1/2 (50.0)	1/17 (5.9)	0/2 (0.0)	0/17 (0.0)	0/2 (0.0)

[a]Unless otherwise stated, all outcomes are presented in frequency and percentages (n/N; (%)). **AE,** adverse event; **IM,** intramuscular; **NA,** not available; **NR,** not reported; **SC,** subcutaneous.

Table S5. Pharmacokinetic profiles of long-acting injectable cabotegravir after the final injection

Arm	Sex; Interval	Cycle (Week)	Author/Trial name	N	Cmax (µg/ml)	Tmax (days); median (range)	C$_τ$ (µg/ml)	AUC0–τ (h× µg/ml)	AUC0-~(h× µg/ml)	$t_{1/2}$ (days)	CL/F (L/h)	>4x PA-IC$_{90}$[b]; n (%)
CAB-LA 800 mg IM	Overall; Single dose 2 x 400 mg	- (>12)	Spreen et al (NCT01756131)(Spreen et al., 2014a)	6	3.3 (75.1)	7.58 (5.0–147.0)	NA	5651 (17.5)	5872 (12.6)	25.4 (51.7)	0.1 (12.5)	NA
	Overall; Q4W	4 (12)	Spreen et al (NCT01593046)(Spreen et al., 2014b)	10	3.3 (59.0)	15.0 (6.0-42.0)	1.1 (140.0)	4467 (52.0)	NA	NA	NA	NA
	Male; Q12W	1 (5)	HPTN 077 (NCT02178800)(Landovitz et al., 2018b)	25	1.89	NA	0.95	2612.6	NA	NA	NA	11 (28.2); n=39
			ECLAIR (NCT02076178)(Markowitz et al., 2017)	93	4.26 (3.64-4.98)	NA	0.30 (0.24-0.39)	3415 (3140-3714)	NA	18.4 (16.4-20.7); n=61	NA	26 (31.0); n=84
		2 (17)	HPTN 077 (NCT02178800)(Landovitz et al., 2018b)	23	2.57	NA	0.78	3197.8	NA	NA	NA	25 (64.1); n=39
			ECLAIR (NCT02076178)(Markowitz et al., 2017)	89	5.22 (4.52-6.04)	NA	0.33 (0.25-0.44); n=71	3873 (3543-4235)	NA	NA	NA	26 (37.1); n=70

		3 (29)	ECLAIR (NCT02076178)(Markowitz et al., 2017)	85	4.91 (4.31-5.60)	NA	0.39 (0.30-0.51); n=66	NA	4047 (3758-4357)	40.0 (35.1-45.7); n=51	0.197 (0.181-0.214); n=68	20 (30.3); n=66
			ECLAIR; Tail-phase (NCT02076178)(Markowitz et al., 2017)		NA	NA	NA	NA	NA	NA	NA	wk12: 20 (23.3); n=86 wk24: 4 (4.7); n=85 ≥wk36: 0 (0.0)
			HPTN 077 (NCT02178800)(Landovitz et al., 2018b)	22	3.39	NA	0.82	4428.2	NA	NA	NA	25 (67.6); n=37
			HPTN 077; Tail-phase (NCT02178800)(Landovitz et al., 2020)	60	NA	NA	NA	NA	NA	45.3 (37.6-54.5)	NA	wk12: 31 (73.8); n=42 wk24: 10 (24.4); n=41 wk36: 2 (5.3); n=38 ≥wk48: 0 (0.0)
	Female; Q12W	1 (5)	HPTN 077 (NCT02178800)(Landovitz et al., 2018b)	49	2.67	NA	0.49	2833.4	NA	NA	NA	37 (75.5)
		2 (17)	HPTN 077 (NCT02178800)(Landovitz et al., 2018b)	39	2.29	NA	1.35	3668.6	NA	NA	NA	37 (94.9); n=39

		3 (29)	HPTN 077 (NCT02178800)(Landovitz et al., 2018b)	35	3.01	NA	1.65	4996.6	NA	NA	NA	35 (100)
			HPTN 077; Tail-phase (NCT02178800)(Landovitz et al., 2020)	117	NA	NA	NA	NA	NA	60.4 (52.9-69.0)	NA	wk12: 83 (97.6); n=85 wk24: 44 (55.0); n=80 wk36: 12 (14.6); n=82 wk48: 2 (2.4); n=82 ≥wk52: 0 (0.0)
CAB-LA 600 mg IM	Male; Q8W	1 (5)	HPTN 077 (NCT02178800)(Landovitz et al., 2018b)	20	2.51	NA	1.79	1227.6	NA	NA	NA	19 (95.0)
		2 (9)		20	3.90	NA	1.29	3518.2	NA	NA	NA	16 (80.0)
		3 (17)		20	2.96	NA	1.11	2994.2	NA	NA	NA	16 (80.0)
		4 (25)		19	2.96	NA	1.46	2917.7	NA	NA	NA	16 (84.2)
		5 (33)		18	3.82	NA	1.68	3913.0	NA	NA	NA	16 (88.9)
	Female; Q8W	1 (5)		39	1.58	NA	1.33	771.8	NA	NA	NA	31 (79.5)
		2 (9)		39	2.96	NA	1.82	3001.2	NA	NA	NA	37 (94.9)
		3 (17)		37	3.46	NA	2.04	3751.0	NA	NA	NA	36 (97.3)
		4 (25)		36	3.33	NA	2.06	3577.0	NA	NA	NA	36 (100)
		5 (33)		32	3.66	NA	2.03	4027.9	NA	NA	NA	31 (96.9)
CAB-LA 400 mg IM	Overall; Single dose 2 x 200 mg	- (>12)	Spreen et al (NCT01756131)(Spreen et al., 2014a)	8	1.40 (53.4)	13.0 (4.0-84.2)	NA	2445 (52.7)	2687 (53.0)	38.3 (57.3); n=4	0.1 (53.0)	NA

	Overall; Single dose 1 x 400 mg	- (>12)		14	0.70 (55.2)	69.0 (2.0-213.0)	NA	1921 (60.9)	2652 (29.8); n=4	31.7 (61.8); n=7	0.1 (29.8); n=4	NA
	Overall; Q4W	4 (12)	Spreen et al (NCT01593046)(Spreen et al., 2014b)	9	4.40 (31.0)	6.0 (2.0-13.1)	3.27 (27.0)	2473 (26.0)	NA	NA	NA	NA
CAB-LA 200 mg IM	Overall; Single dose	- (>12)	Spreen et al (NCT01756131)(Spreen et al., 2014a)	6	0.30 (28.9)	44.5 (27.0-170.0)	NA	1068 (37.4)	1234 (34.6)	53.9 (32.2)	0.1 (34.6)	NA
	Overall; Q4W	4 (12)	Spreen et al (NCT01593046)(Spreen et al., 2014b)	8	2.20 (31.0)	6.0 (2.0-27.0)	1.61 (64.0)	1242 (39.0)	NA	NA	NA	NA
CAB-LA 100 mg IM	Overall; Single dose	- (>12)	Spreen et al (NCT01756131)(Spreen et al., 2014a)	6	0.20 (58.6)	9.0 (4.0-83.0)	NA	607 (43.3)	920 (12.3)	33.3 (66.8)	0.1 (12.3); n=10	NA
CAB-LA 400 mg SC	Overall; Single dose 2 x 200 mg	- (>12)	Spreen et al (NCT01756131)(Spreen et al., 2014a)	10	0.9 (83.4)	27.0 (3.0-83.0)	NA	2402 (16.1)	2734 (22.3)	42.8 (52.0)	0.1 (22.3); n=6	NA
CAB-LA 200 mg SC	Overall; Single dose	- (>12)	Spreen et al (NCT01756131)(Spreen et al., 2014a)	10	0.5 (48.6)	6.0 (3.0-27.0)	NA	1005 (85.7)	1706 (2.6); n=4	42.7 (53.1); n=4	0.1 (2.6)	NA
	Overall; Q4W	4 (12)	Spreen et al (NCT01593046)(Spreen et al., 2014b)	9	2.10 (21.0)	6.0 (2.0-13.0)	1.66 (31.0)	1244 (23.0)	NA	NA	NA	NA

CAB-LA 100 mg SC	Overall; Single dose	- (>12)	Spreen et al (NCT01756131)(Spreen et al., 2014a)	6	0.20 (62.1)	16.50 (4.0-55.0)	NA	433 (102.0)	689 (24.0); n=5	50.4 (76.9); n=5	0.1 (24); n=5	NA

[a]Unless explicitly stated, all outcomes are presented in geometric mean (95% confidence interval). [b]4x PA-IC_{90} of CAB-LA is 0.664 µg/mL.(Markowitz et al., 2017; Landovitz et al., 2018b) **$AUC_{0-τ}$,** area under the plasma drug concentration at τ weeks; **$AUC_{0-\infty}$,** area under the plasma drug concentration; **CAB,** cabotegravir; **CI,** confidence interval; **Cmax,** maximum plasma concentration; **Cτ,** plasma concentration at τ weeks; **GM,** geometric mean; **IM, intramuscular; LA,** long acting; **RPV,** rilpivirine; **SC,** subcutaneous; **SD,** standard deviation; **$t_{1/2}$,** apparent half-life; **Tmax,** time needed to reach Cmax.

Table S6. Pharmacokinetic profiles of long-acting injectable rilpivirine after the final injection

Arm	Sex; Interval	Cycle (Week)	Author/Trial name	N	Cmax (ng/ml)	Tmax (days); median (range)	C_{τ} (μg/ml)	$AUC_{0–\tau}$ (h× ng/ml)	$AUC_{0-\infty}$ (h× μg/ml)	$t_{1/2}$ (days)	>4 x PA-IC_{90}[b]; n (%)
RPV-LA 1200/600/600 mg IM	Overall; Q4W	1 (1)	Verloes et al (NCT01031589)(Verloes et al., 2015)	6	Mean ± SD: 140 ± 16	3.0 (2.0-5.0)	NA	Mean ± SD: 55,350 ± 13,550	NA	NA	NA
		2 (5)		5	Mean ± SD: 120 ± 40	2.0 (1.0-3.0)	Mean ± SD: 63.0 ± 17.0	Mean ± SD: 47,420 ± 15,830	NA	NA	NA
		3 (9)		5	Mean ± SD: 132 ± 19	2.0 (2.0-3.0)	Mean ± SD: 53.0 ± 20.0	Mean ± SD: 52,810 ± 13,070	NA	Mean ± SD: 91 ± 14	NA
RPV-LA 1200 mg IM	Female; Single dose	- (12)	Jackson et al (NCT01275443)(Jackson et al., 2014)	20	160 (132.6-187.4)	GM 6.2 (95% CI: 3.9-8.5)	NA	143,568 (119,947.2-167,188.8)	NA	38.0 (28.5-47.5)	NA
	Overall; Q8W	1 (4)	HPTN 076 (NCT02165202)(Bekker et al., 2020)	80	NA	NA	38.0 (26.0-55.5)	NA	NA	NA	19 (29.7); n=64
		1 (6)			NA	NA	94.4 (82.7-107.8)	NA	NA	NA	NA
		1 (8)			NA	NA	73.3 (64.8-82.9)	NA	NA	NA	NA
		2 (12)			NA	NA	40.2 (37.1-43.6)	NA	NA	NA	49 (76.5); n=64

		2 (14)			NA	NA	116.3 (103.0-131.4)	NA	NA	NA	NA
		3 (20)			NA	NA	57.4 (51.2-64.2)	NA	NA	NA	57 (89.0); n=64
		4 (38)			NA	NA	64.9 (56.8-74.1)	NA	NA	NA	56 (88.9); n=63
		5 (36)			NA	NA	71.4 (61.-83.2)	NA	NA	NA	60 (93.8); n=64
		6 (44)			NA	NA	72.5 (61.2-85.9)	NA	NA	NA	61 (98.4); n=62
		6 (52)			NA	NA	75.0 (61.6-91.4)	NA	NA	NA	NA
RPV-LA 900 mg IM	Overall; Q4W	4 (12)	Spreen et al (NCT01593046)(Spreen et al., 2014b)	8	168 (37.0)	6.0 (2.0-13.0)	79.1 (44.0)	74,420 (35.0)	NA	NA	NA
RPV-LA 600 mg IM	Overall; Q4W	4 (12)	Spreen et al (NCT01593046)(Spreen et al., 2014b)	9	126 (32)	9.5 (2.0-27.0)	78.9 (39.0)	63,656 (34.0)	NA	NA	NA
	Overall; Single dose	- (4)	Verloes et al (NCT01031589)(Verloes et al., 2015)	5	Mean ± SD: 48 ± 13	9.0 (5.0-28.0)	NA	Mean ± SD: 25,240 ± 8184	NA	Mean ± SD: 61 ± 20	NA

	Female; Single dose	- (12)	Jackson et al (NCT01275443)(Jackson et al., 2014)	20	82 (66.5-97.5)	GM 6 (95% CI: 2.9-9.1)	NA	70,416 (59,978.4-80,853.6)	NA	39.0 (31.9-46.1)	NA
	Male; Single dose	- (12)	Jackson et al (NCT01275443)(Jackson et al., 2014)	20	114.1 (84.0-144.2)	GM 5 (95% CI: 0-11.0)	NA	92,961.6 (76,099.2-109,824.0)	NA	30.5 (25.3-35.7)	NA
RPV-LA 300 mg IM	Overall; Single dose	- (4)	Verloes et al (NCT01031589)(Verloes et al., 2015)	6	Mean ± SD: 39 ± 25	11.5 (2.0-22.0)	NA	Mean ± SD: 17,090 ± 8907	NA	Mean ± SD: 44 ± 24	NA

[a]Unless otherwise specified, all outcomes are presented in geometric mean (95% confidence interval) or geometric mean (%CV). [b]4x PA-IC_{90} of RPV-LA is 50 ng/mL.(Bekker et al., 2020)

%CV, between-person coefficient of variation; **$AUC_{0-\tau}$,** area under the plasma drug concentration at τ weeks; **$AUC_{0-\infty}$,** area under the plasma drug concentration; **CAB,** cabotegravir; **CI,** confidence interval; **Cmax,** maximum plasma concentration; **Cτ,** plasma concentration at τ weeks; **GM,** geometric mean; **IM, intramuscular; LA,** long acting; **RPV,** rilpivirine; **SC,** subcutaneous; **SD,** standard deviation; **$t_{1/2}$,** apparent half-life; **Tmax,** time needed to reach Cmax.

REFERENCES

Bekker, L. G., Li, S., Pathak, S., Tolley, E. E., Marzinke, M. A., Justman, J. E., et al. (2020). Safety and tolerability of injectable Rilpivirine LA in HPTN 076: A phase 2 HIV pre-exposure prophylaxis study in women. *EClinicalMedicine* 21, 100303. doi:10.1016/j.eclinm.2020.100303.

Benet, L. (1984). Pharmacokinetic parameters: which are necessary to define a drug substance? - PubMed. *Eur J Respir Dis Suppl* 134, 45–61.

Grabowski, T., Jaroszewski, J. J., Piotrowski, W., and Sasinowska-Motyl, M. (2014). Method of variability optimization in pharmacokinetic data analysis. *Eur. J. Drug Metab. Pharmacokinet.* 39, 111–119. doi:10.1007/s13318-013-0145-x.

Higgins, J. P. T., Thomas, J., Chandler, J., Cumpston, M., Li, T., Page, M. J., et al. eds. (2019). *Cochrane handbook for systematic reviews of interventions version 6.0 (updated July 2019).* 6th ed. Cochrane doi:10.1002/9781119536604.

Jackson, A. G. A., Else, L. J., Mesquita, P. M. M. M., Egan, D., Back, D. J., Karolia, Z., et al. (2014). A compartmental pharmacokinetic evaluation of long-acting rilpivirine in HIV-negative volunteers for pre-exposure prophylaxis. *Clin. Pharmacol. Ther.* 96, 314–323. doi:10.1038/clpt.2014.118.

Landovitz, R. J., Li, S., Eron, J. J., Grinsztejn, B., Dawood, H., Liu, A. Y., et al. (2018a). Tail-phase safety, tolerability and pharmacokinetics of long-acting injectable cabotegravir in hiv-uninfected individuals: HPTN 077 final results. *AIDS Res. Hum. Retroviruses* 34, 57--.

Landovitz, R. J., Li, S., Eron, J. J., Grinsztejn, B., Dawood, H., Liu, A. Y., et al. (2020). Tail-phase safety, tolerability, and pharmacokinetics of long-acting injectable cabotegravir in HIV-uninfected adults: a secondary analysis of the HPTN 077 trial. *Lancet HIV* 7, e472–e481. doi:10.1016/S2352-3018(20)30106-5.

Landovitz, R. J., Li, S., Grinsztejn, B., Dawood, H., Liu, A. Y., Magnus, M., et al. (2018b). Safety, tolerability, and pharmacokinetics of long-acting injectable cabotegravir in low-risk HIV-uninfected individuals: HPTN 077, a phase 2a randomized controlled trial. *PLoS Med.* 15, e1002690--e1002690. doi:10.1371/journal.pmed.1002690.

Lazarus, G., and Christianto Safety, tolerability, and pharmacokinetics profile of long-acting injectable pre-exposure prophylaxis for Human Immunodeficiency Virus prevention: a systematic review and meta-analysis of clinical trials. PROSPERO 2020. CRD42020154772. Available at: https://www.crd.york.ac.uk/PROSPERO/display_record.php?RecordID=154772 [Accessed November 11, 2020].

Markowitz, M., Frank, I., Grant, R. M., Mayer, K. H., Elion, R., Goldstein, D., et al. (2017). Safety and tolerability of long-acting cabotegravir injections in HIV-uninfected men (ECLAIR): a multicentre, double-blind, randomised, placebo-controlled, phase 2a trial. *lancet. HIV* 4, e331--e340. doi:10.1016/S2352-3018(17)30068-1.

National Institute of Allergy and Infectious Diseases (2017). Division of AIDS (DAIDS) table for grading the severity of adult and pediatric adverse events, corrected version 2.1. doi:10.7326/0003-4819-157-11-201212040-00003.

Spreen, W., Ford, S. L., Chen, S., Wilfret, D., Margolis, D., Gould, E., et al. (2014a). GSK1265744 pharmacokinetics in plasma and tissue after single-dose long-acting injectable administration in healthy subjects. *J. Acquir. Immune Defic. Syndr.* 67, 481–486. doi:10.1097/QAI.0000000000000301.

Spreen, W., Williams, P., Margolis, D., Ford, S. L., Crauwels, H., Lou, Y., et al. (2014b). Pharmacokinetics, safety, and tolerability with repeat doses of GSK1265744 and rilpivirine (TMC278) long-acting nanosuspensions in healthy adults. in *Journal of Acquired Immune Deficiency Syndromes* (Lippincott Williams and Wilkins), 487–492. doi:10.1097/QAI.0000000000000365.

Sterne, J. A. C., Savović, J., Page, M. J., Elbers, R. G., Blencowe, N. S., Boutron, I., et al. (2019). RoB 2: a revised tool for assessing risk of bias in randomised trials. *BMJ* 366, I4898.

Urso, R., Blardi, P., and Giorgi, G. (2002). A short introduction to pharmacokinetics. *Eur. Rev. Med. Pharmacol. Sci.* 3, 33–44.

Verloes, R., Deleu, S., Niemeijer, N., Crauwels, H., Meyvisch, P., and Williams, P. (2015). Safety, tolerability and pharmacokinetics of rilpivirine following administration of a long-acting formulation in healthy volunteers. *HIV Med.* 16, 477–484. doi:10.1111/hiv.12247.
